# Supplementary material for: Distinctive Toll-like Receptors Gene Expression and Glial Response in Different Brain Regions of Natural Scrapie
Source: Int J Mol Sci. 2022 Mar 25;23(7):3579. doi: 10.3390/ijms23073579 (PMC8998348; doi:10.3390/ijms23073579)
Supplement: Supplementary file 1 [file ijms-23-03579-s001.zip › ijms-1652083-supplementary.pdf]

Supplementary materials

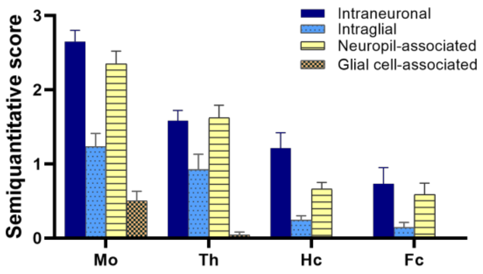

Figure S1. Types of PrP^{Sc} patterns observed in scrapie-infected sheep in the medulla oblongata (Mo), thalamus (Th), hippocampus (Hc) and frontal cortex (Fc).

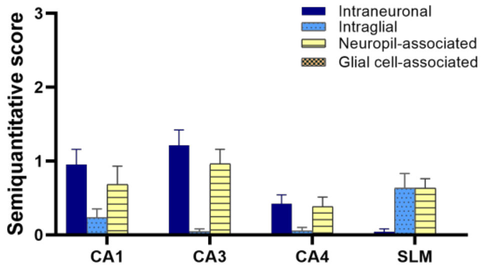

Figure S2. Types of PrP^{Sc} patterns observed in the four regions of the hippocampus: CA1, CA3, and CA4 pyramidal cell layers and *stratum lacunosum-moleculare (SLM)*. Scores range from 0 (absent), 1 (mild), 2 (moderate), and 3 (severe).

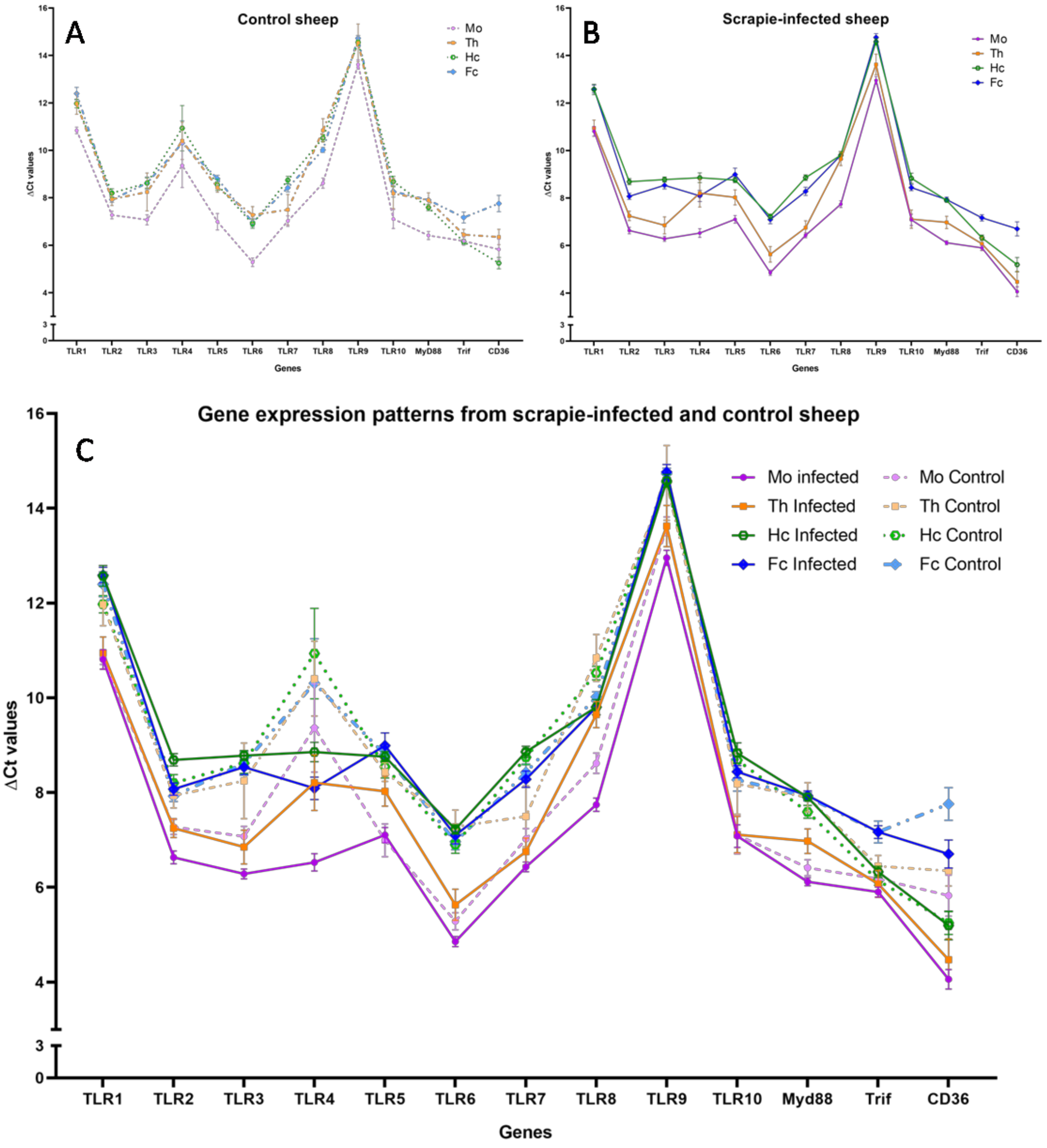

Figure S3. ΔCt values of the medulla oblongata (Mo), thalamus (Th), hippocampus (Hc), and frontal cortex (Fc) of control sheep **(A)**, scrapie-infected sheep **(B)** and comparing both groups **(C)**. Dara are represented as the mean ±SEM.

Table S1. Summary of qPCR results of the genes that displayed *p-values <0.1.* Fold-change values were calculated with the $2^{\wedge-\Delta\Delta Ct}$ method and values from the control group were scaled to 1. Fold-change mean scores were compared using the Student's t-test or Mann-Whitney U test depending on the parametric o no parametric data distribution.

Specie	Brain area	Gen	Fold change	*p-value*
**Sheep**	Medulla oblongata	*TLR2*	1.56	0.009
		TLR3	1.73	0.001
		TLR4	7.16	0.012
		TLR6	1.35	0.036
		TLR7	1.5	0.013
		TLR8	1.83	0.002
		TLR9	1.59	0.011
		CD36	3.40	0.001
	Thalamus	*TLR2*	1.61	0.075
		TLR3	2.63	0.082
		TLR6	3.13	0.011
		MyD88	2.33	0.001
		CD36	3.67	0.007
		TGF-β	2.28	0.002
		IL-10	2.59	0.003
		IL-6	1.91	0.032
	Hippocampus	*TLR1*	0.66	0.073
		TLR2	0.71	0.035
		MyD88	0.81	0.043
	Frontal cortex	*TLR4*	4.66	0.023
		CD36	2.08	0.035
**Tg338**	Thalamus	*TLR1*	1.69	0.001
		TLR2	1.63	0.005
		TLR7	1.31	0.065
